# Expression, localisation and potential significance of aquaporins in benign and malignant human prostate tissue

**DOI:** 10.1186/s12894-018-0391-y

**Published:** 2018-09-03

**Authors:** Johannes Bründl, Sabine Wallinger, Johannes Breyer, Florian Weber, Matthias Evert, Nikolaos Theodoros Georgopoulos, Bernd Rosenhammer, Maximilian Burger, Wolfgang Otto, Peter Rubenwolf

**Affiliations:** 1 Department of Urology, Caritas St Josef Medical Center, University of Regensburg, Landshuter Straße 65, 93053 Regensburg, Germany; 2 Institute of Pathology, University of Regensburg, Regensburg, Germany; 3 Department of Biological Sciences, School of Applied Sciences, University of Huddersfield, Huddersfield, UK; 4 Department of Urology, Frankfurt University Medical Center, Frankfurt, Germany

**Keywords:** Aquaporins, Human prostate, Prostate cancer, Prostate cancer cell lines

## Abstract

**Background:**

To study the expression pattern, localisation and potential clinical significance of aquaporin water channels (AQP) both in prostate cancer (PC) cell lines and in benign and malignant human prostate tissue.

**Methods:**

The AQP transcript and protein expression of HPrEC, LNCaP, DU-145 and PC3 cell lines was investigated using reverse transcriptase polymerase chain reaction (RT-PCR) and immunofluorescence (IF) microscopy labelling. Immunohistochemistry (IHC) was performed to assess AQP protein expression in surgical specimens of benign prostatic hyperplasia as well as in PC. Tissue mRNA expression of AQPs was quantified by single-step reverse transcriptase quantitative polymerase chain reaction (qPCR). Relative gene expression was determined using the 40-ΔC_T_ method and correlated to clinicopathological parameters.

**Results:**

Transcripts of AQP 1, 3, 4, 7, 8, 10 and 11 were expressed in all four cell lines, while AQP 9 transcripts were not detected in malignant cell lines. IF microscopy confirmed AQP 3, 4, 5, 7 and 9 protein expression. IHC revealed highly heterogeneous AQP 3 protein expression in PC specimens, with a marked decrease in expression in tumours of increasing malignancy. Loss of AQP 9 was shown in PC specimens. mRNA expression of AQP3 was found to be negatively correlated to PSA levels (*ρ* = − 0.354; *p* = 0.013), D’Amico risk stratification (*ρ* = − 0.336; *p* = 0.012), ISUP grade (*ρ* = − 0.321; *p* = 0.017) and Gleason score (ρ = − 0.342; *p* = 0.011).

**Conclusions:**

This is the first study to systematically characterize human prostate cell lines, benign prostatic hyperplasia and PC in relation to all 13 members of the AQP family. Our results indicate the differential expression of several AQPs in benign and malignant prostate tissue. A significant correlation was observed between AQP 3 expression and tumour grade, with progressive loss in more malignant tumours. Taken together, AQPs may play a role in the progression of PC and AQP expression patterns may serve as a prognostic marker.

**Electronic supplementary material:**

The online version of this article (10.1186/s12894-018-0391-y) contains supplementary material, which is available to authorized users.

## Background

Water and solute movement across the epithelia lining the male reproductive tract are essential prerequisites for seminal fluid formation and homeostasis, and are of paramount significance for the modulation of the luminal environment in which sperm cells mature and reside.

The mechanism by which water crosses through epithelial borders had remained a matter of debate until the discovery and elucidation of the function of the aquaporin water channels (AQP) by the later Nobel laureate Peter Agre in the early 1990s. AQPs are a family of transmembrane pore-forming proteins that selectively allow water and other small, uncharged molecules such as urea, glycerol and pyrimidines to pass along hydrostatic and osmotic gradients. They play a fundamental role in numerous physiological processes, most notably in fluid absorption and secretion. To date, 13 different mammalian AQPs have been identified at the molecular level and localised in specific tissues [1]. Analysis of several human diseases has confirmed that AQPs are functionally involved in various pathological conditions and thus may provide promising drug targets [2]. Moreover, there is strong presumptive evidence that AQPs play a role in carcinogenesis, specifically in tumour angiogenesis and cell migration [3].

To date, the presence and significance of AQPs in the human prostate remain largely uninvestigated, with reports of the individual expression of AQP 1, 3, 5 and 9 documented in previous studies [4–8]. These findings suggest that fluid reabsorption and secretion in the prostate could be modulated by AQPs. Yet, despite investigations examining expression for individual AQPs, the human prostate has not been systematically studied in relation to all 13 members of the AQP family.

The principal aim of this study was to systematically characterize the expression pattern of all 13 AQP channels in cultured normal and malignant prostate epithelial cells, as well as freshly-isolated benign and malignant human prostate tissues. This approach allowed us to systematically study expression both at the mRNA and protein level of the whole AQP family in prostate tissue, and to correlate the pattern of expression with clinicopathological parameters. The potential biological and clinical significance of our findings are discussed.

## Methods

### Human prostate cell lines

#### Cell culture

Four established human prostate cell lines, one normal human prostate epithelial cell line (HPrEC, Lifeline Cell Technology, USA), and three cancer cell lines, PC3 (Lifeline Cell Technology, USA), DU145 and LNCaP (CLS Cell Lines Service GmbH, Germany) were grown to confluency under standard culture conditions as follows: HPrEC: ProstaLife™ Basal Medium + ProstaLife™ LifeFactors Kit (37 °C; 5%CO_2_); LNCaP/DU-145/PC3: DMEM + RPMI (1:1), 5% FBS (fetal bovine serum), 1% L-Glutamine (37 °C; 5%CO_2_).

### Ribonucleic acid isolation and transcript analysis (RT-PCR)

Confluent cultures of prostate cell lines were rinsed twice in phosphate-buffered saline (PBS) and harvested for the isolation of total RNA according to the manufacturer’s recommendations. RNA was extracted using RNeasy Mini Kit (Qiagen) after incubation with Proteinase K for 10 min. RNA quantity was assessed using a Nanodrop spectrometer (Nanodrop 2000c, Thermo Scientific). RNA from cell lines was reverse transcribed to cDNA using the iScript cDNA Synthesis Kit (Biorad) in line with the manufacturer’s protocol. AQP 0–12 primers were designed using the National Centre for Biotechnology Information (NCBI) database resources (primer sequences specified in Additional file 1: Table S1) with ß-actin serving as the transcript control. RT-PCR conditions were as follows: 3 min at 95 °C, followed by 30 cycles at 95 °C for 10 s, annealing for 30 s (specific annealing temperatures specified in Additional file 1: Table S1), extension at 72 °C for 30 s, and 8 min elongation at 72 °C. PCR products were analysed with 2% agarose gel electrophoresis exactly as described in [9]. Reverse transcriptase (RT)-positive and RT-negative controls were included in all PCR reactions [9, 10].

### Immunofluorescence (IF) microscopy

Cells from all four human prostate cell lines were seeded at 1 × 10^5^ cells/ml onto Multiwell glass slides. Cultures were fixed in a 1:1 (*v*/v) solution of methanol and acetone, air-dried, and incubated sequentially with the primary antibody for 16 h at 4 °C (see Additional file 2: Table S2) and the secondary antibody conjugated with Alexa 594 (Molecular Probes) for 30 min, with PBS washing between steps. Hoechst 33258 (DAPI; 0.1 mg/ml) was used to stain nuclei. Secondary antibody-only negative controls and positive control cell lines known to express the respective antigen were included as specificity controls [9, 10]. Immunolabelling was visualised by epifluorescence on a Zeiss Axio Imager Z1 microscope.

### Human prostate specimens

#### Collection of specimens

The collection of tissues had the approval of the local research ethics committee (reference number: 17–660-101) and written patient consent was also obtained. Human prostate samples were obtained from 61 patients who underwent a suprapubic adenomectomy for benign prostatic hyperplasia (BPH, *n* = 15) or a robot-assisted radical prostatectomy for biopsy-proven prostate cancer (*n* = 46) at our department in 2014. None of these patients had undergone hormonal therapy prior to surgery.

### Clinicopathological data

Clinicopathological data are summarized in Table 1. All surgical specimens were assessed histopathologically by two independent uropathologists for grading and staging based on the criteria of the 2009 UICC TNM classification and ISUP 2014 Gleason grade groups and subsequently prepared for microdissection [11].

**Table 1:** Patient characteristics

	*n* (%)
Patient data	
Age (years; range)	66 (47–84)
Total number	61 (100%)
Benign prostatic hyperplasia (BPH)	15 (24.6%)
Low-risk PC (D’Amico)	16 (26.2%)
Intermediate PC (D’Amico)	16 (26.2%)
High-risk PC (D’Amico)	14 (23.0%)
PSA	
< 4 ng/ml	2 (3.3%)
4-10 ng/ml	36 (59.0%)
10-20 ng/ml	12 (19.7%)
> 20 ng/ml	5 (8.2%)
n/a	6 (9.8%)
ISUP (Gleason-Score)	
1 (6)	17 (27.9%)
2 (7a)	14 (23.0%)
3 (7b)	1 (1.6%)
4 (8)	3 (4.9%)
5 (9–10)	11 (18.0%)
No cancer	15 (24.6%)
T-stage	
pT2a	7 (11.5%)
pT2b	1 (1.6%)
pT2c	28 (45.9%)
pT3/4	10 (16.4%)
No cancer	15 (24.6%)

### Ribonucleic acid isolation

Paraffin wax-embedded samples were de-paraffinized in xylene and microdissected samples from five serial sections (10 μm) were pooled and collected in 50 ml of lysis buffer (Qiagen). RNA was extracted using a FFPE RNA Kit (Qiagen).

### Real-time quantitative polymerase chain reaction (qPCR)

RNA from microdissected tissue was reverse transcribed and amplified using the iTaq Universal SYBR Green One-Step Kit (Biorad) and real-time PCR reaction was carried out on a CFX Connect Real-Time PCR Detection System (Biorad) using SYBR-Green I chemistry. Quantification was performed on MicroAmp Optical 96-Well Reaction plates. Detection of PCR products was accomplished by measuring the emitting fluorescence at the end of each reaction step. Forty amplification cycles were applied and the cycle threshold (C_T_) values of AQP 3, AQP 4, AQP 7, AQP 9 were determined along with one reference gene for each AQP. The SYBR-Green assay module includes a final melting point analysis that followed the 40 cycles of quantitative PCR. Plots from the melting point analysis were manually inspected for all RNA gene assays tested to verify that primers were specific (data not shown). PBGD (porphobilinogen desaminase) was used as housekeeping gene as previously described [12]. C_T_ values were normalized by subtracting the C_T_ value of the housekeeping gene from the C_T_ value of the target gene (ΔC_T_). RNA results were then reported as 40-ΔC_T_ values to ensure that the normalized gene expression obtained by the test was proportional to the corresponding mRNA expression levels [13, 14].

### Immunohistochemistry

Surgical samples were fixed in 10% formalin, dehydrated, and embedded in paraffin wax. Dewaxed 4-μm tissue sections were subjected to antigen retrieval by boiling for 10 min in tris-ethylenediaminetetraacetic acid (Tris-EDTA, pH 9 for AQP 3) or citric acid (pH 6 for AQP 4, 5, 7, and 9), before labelling with pre-titrated primary antibodies (see Additional file 2: Table S2) for 16 h at 4 °C. Secondary antibody-only controls and positive control tissues known to express the respective antigen were included as specificity controls [9, 10].

### Statistical analysis

Statistical analyses were performed using SPSS version 23. The Spearman’s rank correlation coefficient ρ was used as a measure of the strength and direction of the relationship between variables. Levels of mRNA-expression were stratified by quartiles.

## Results

### AQP expression by human prostate cell cultures in vitro

**Fig. 1:**
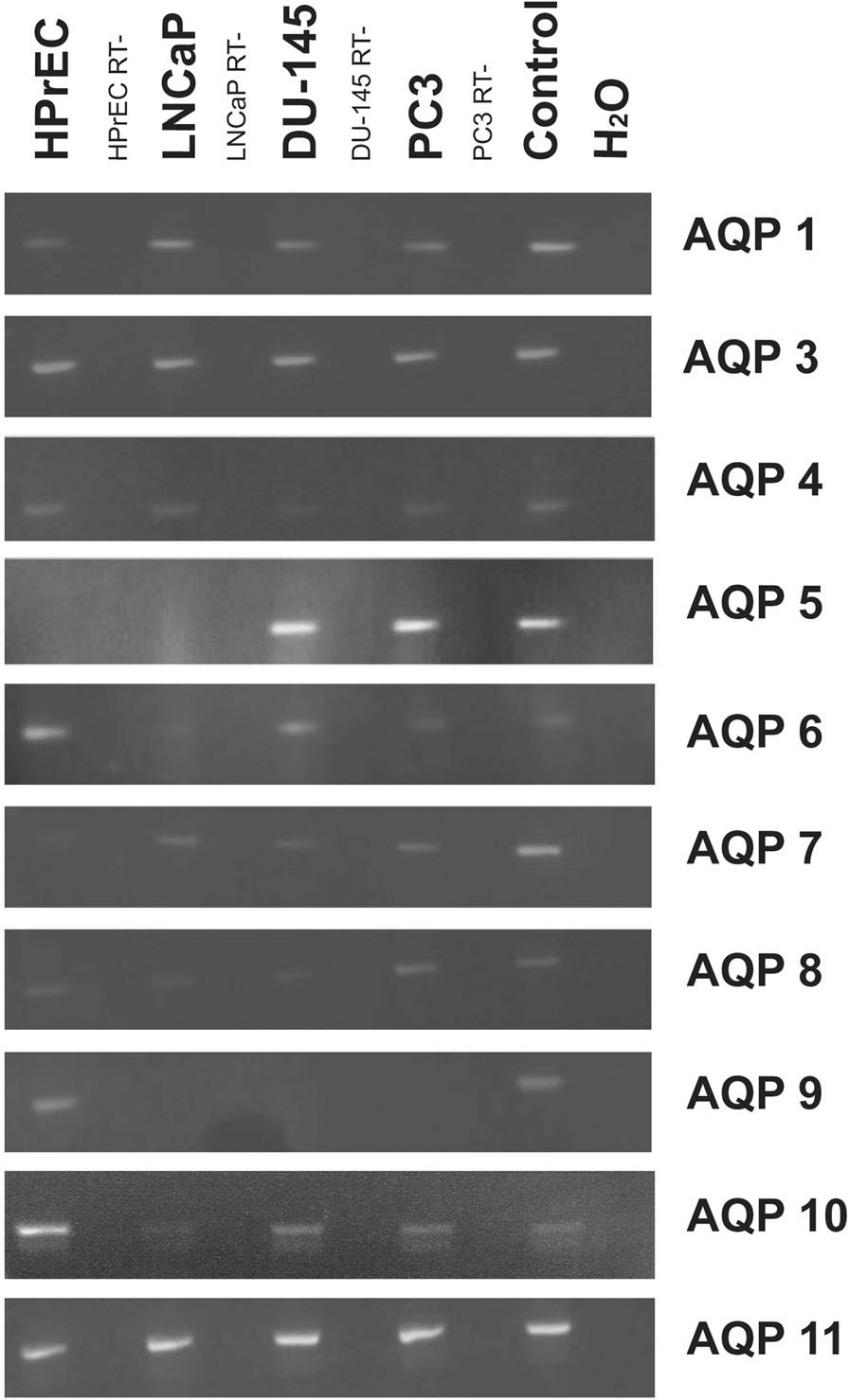
AQP transcript expression: RT-PCR results of AQP 0–12 in normal prostate epithelial cells (HPrEC) and in established PC lines (LNCaP, DU-145 and PC3). AQP 1, 3, 4, 7, 8, 10 and 11 were detected in HPrEC, LNCaP, DU-145 and PC3. AQP 5 was expressed by DU-145 and PC3, but not by HPrEC and LNCaP. Transcripts of AQP 6 were present in all cell lines except in LNCaP. AQP 9 transcripts were found in HPrEC, but not in PC lines. ß-Actin, a no-template control, and positive controls were included. RT-, reverse transcriptase-negative samples

AQP gene expression was investigated by RT-PCR and immunofluorescence microscopy. In vitro, normal human prostate epithelial cells (HPrEC) as well as malignant cell lines (LNCaP, DU-145 and PC3) showed expression of AQP 1, 3, 4, 7, 8, 10 and 11 (see Fig. 1). By contrast, transcripts for AQP 0, 2, and 12 were not detected (not shown). AQP 5 mRNA transcripts were detected in the DU-145 and PC3 cancer cell lines, but not in LNCaP or HPrEC cells. Transcripts of AQP 6 were present in all cell lines except in LNCaP cells. AQP 9 transcripts were found in HPrEC, but not in malignant cell lines.

**Fig. 2:**
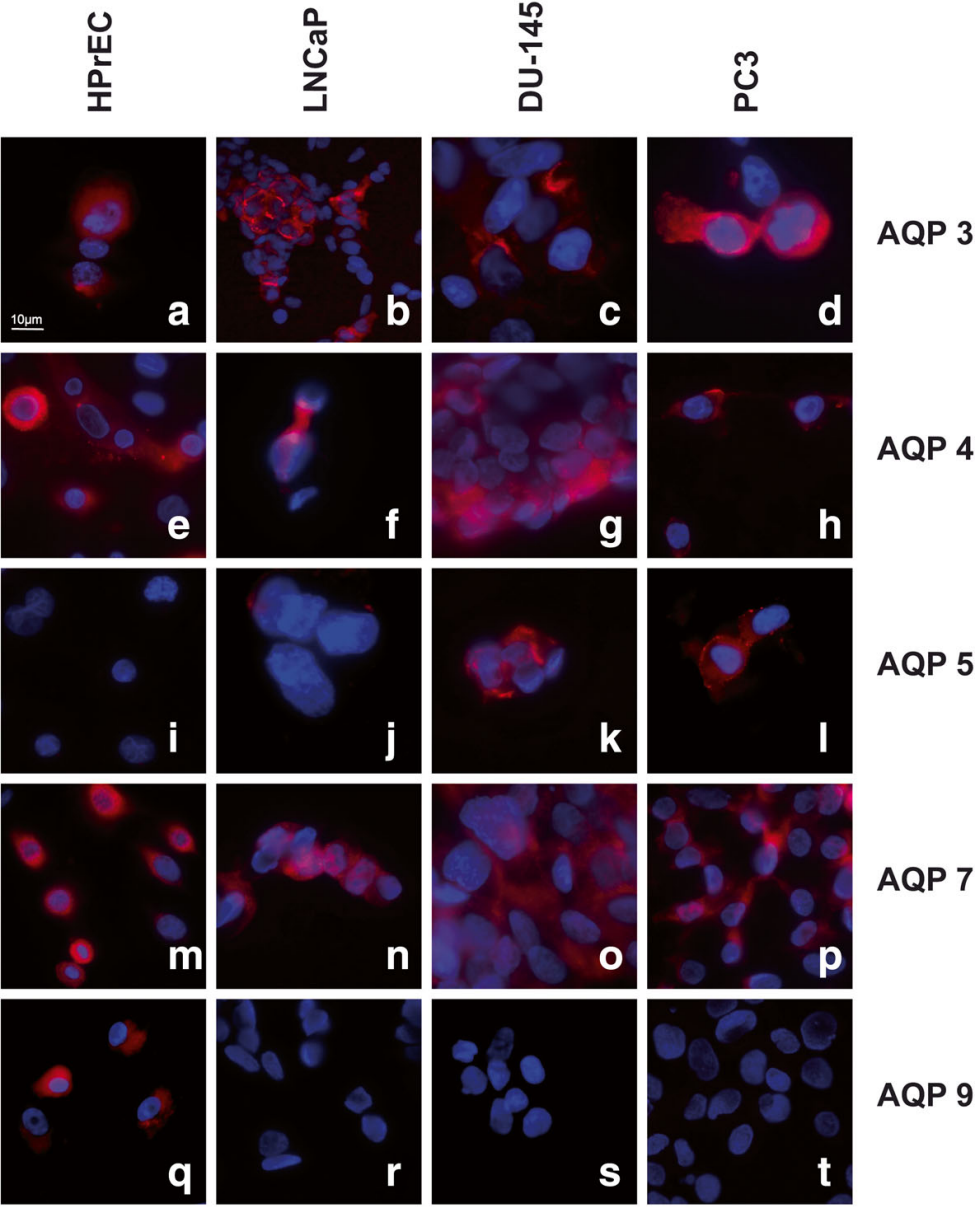
Immunofluorescence labelling of HPrEC, LNCaP, DU-145 and PC3 cell lines. Intense expression of AQP 3 (**a**-**d**) AQP 4 (**e**-**h**) and AQP 7 (**m**-**p**) in all cell lines. No expression of AQP 5 was detected in HPrEC and LNCaP, but positive immunofluorescence labelling in DU-145 and PC3 (**i**-**l**). Expression of AQP 9 in HPrEC with no expression in LNCaP, DU-145 and PC3 (**q**-**t**)

Using immunofluorescence microscopy, AQP 3, 4 and 7 were detected in normal cells as well as the three PC cell lines, whereas AQP 5 was only expressed in DU-145 and PC3 cancer cell lines, in line with the RT-PCR analysis results. Expression of AQP 9 was found in normal prostate cells (HPrEC), but was absent in all three cancer cell lines (LNCaP, DU-145, PC3), also in accordance with the transcript findings. Representative results of these experiments are shown in Fig. 2. The remaining AQPs could not be assessed for protein expression due to the lack of suitable commercially available antibodies.

### AQP expression by native human prostate tissue

**Fig. 3:**
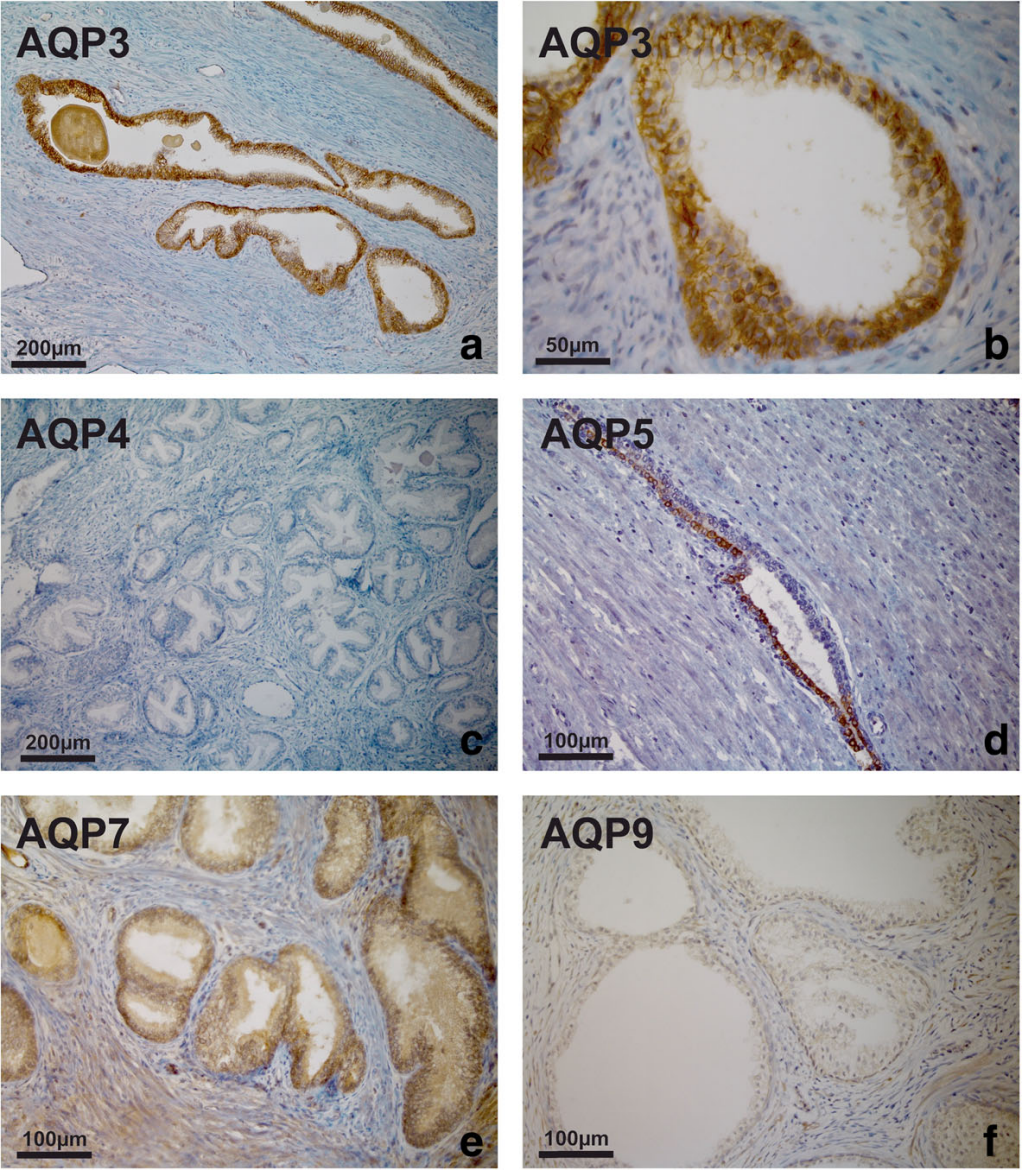
AQP immunoperoxidase labelling of non-malignant prostatic tissues (BPH). Intense expression of AQP 3 throughout the epithelium from a patient with BPH. Labelling was most intense in the basal layer, less intense in the intermediate layer and weak in the luminal cells (**a**). At higher magnification, a distinct localisation pattern was apparent, with intense labelling of intercellular borders in the basal and suprabasal compartments (**b**). No immunoreactivity was seen in the submucosa, smooth muscle or endothelium. No expression of AQP 4 was detected (**c**). Heterogeneous expression of AQP 5 (**d**), diffuse expression of AQP 7 (**e**) and weak expression of AQP 9 (**f**) was observed

Having detected AQP expression at the mRNA and protein level in normal and malignant cell lines in vitro, the level of expression and cellular localisation of AQP protein in native, non-malignant prostate tissue was examined by immunohistochemistry. AQP 3 was strongly expressed throughout the epithelium of all specimens with benign prostatic hyperplasia (BPH). Labelling was most intense in the basal layer, less intense in the intermediate layer and weak in the luminal cells. At higher magnification, a distinct localisation pattern was apparent, with intense labelling at the intercellular borders in the basal and suprabasal compartments, as shown in Fig. 3. No immunoreactivity was seen in the submucosa, smooth muscle or endothelium. AQP 4 was not detected in any BPH specimen. AQP 5, AQP 7 and AQP 9 were found to be present in the epithelium of all benign tissues, although the expression pattern was less unequivocal compared with AQP 3 (Fig. 3).

**Fig. 4:**
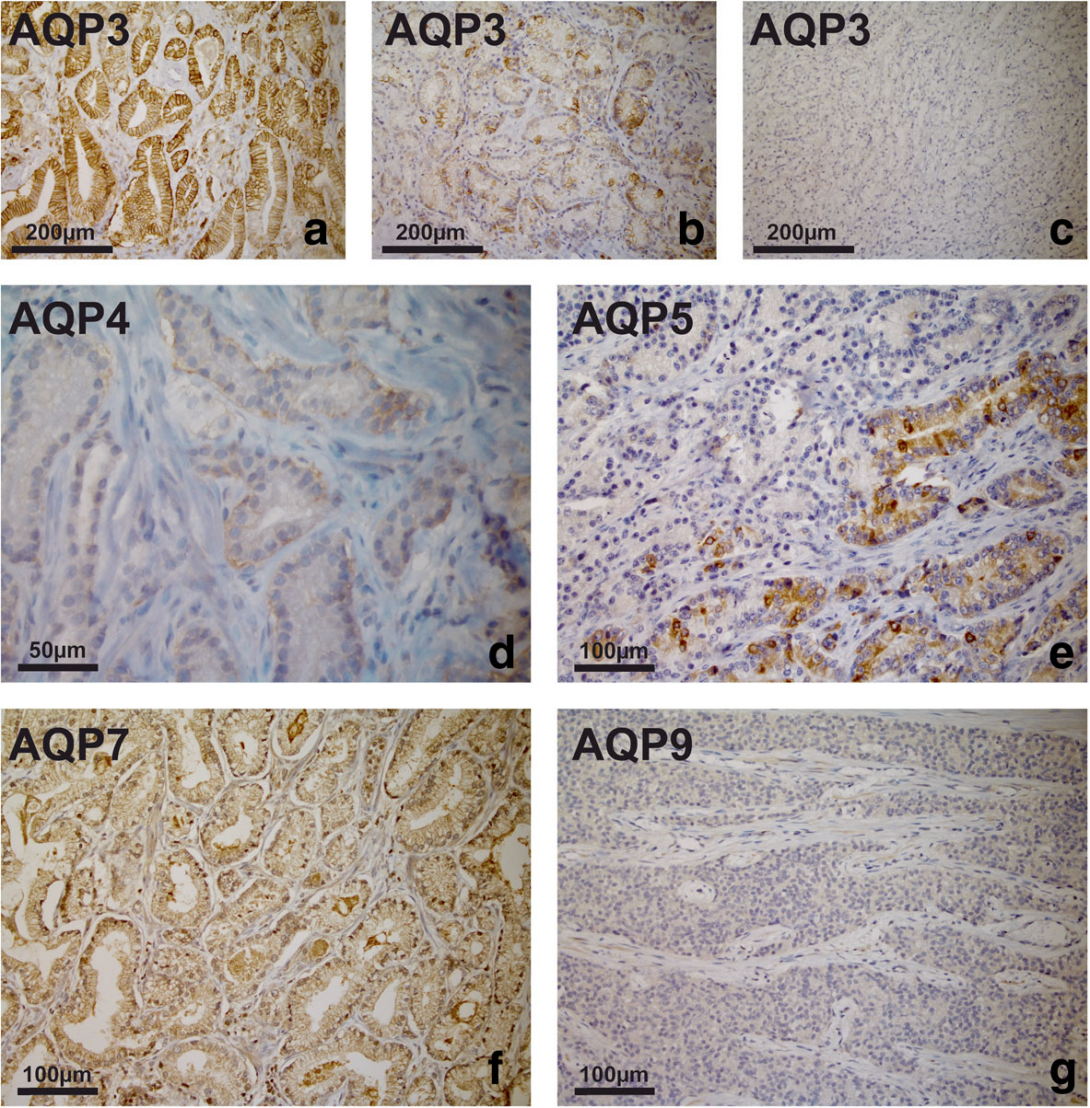
AQP immunoperoxidase labelling of prostate cancer specimens. Intense expression with distinct labelling at the cell borders in a sample of a low-risk tumour (**a**), less intense expression of AQP 3 with alternating AQP-positive and AQP-negative tumour cells found in an intermediate-risk tumour (**b**). Complete loss of AQP 3 expression in a high-risk tumour (**c**). Weak expression of AQP 4 in an intermediate-risk tumour (**d**). Heterogeneous expression of AQP 5 (**e**) and expression of AQP 7 (**f**) in PC. AQP 9 was not detected (**g**)

In contrast to our observations in non-malignant (BPH) tissue, a highly heterogeneous AQP 3 protein expression was observed in prostate cancer specimens, with marked decrease in intensity and expression in tumours of increasing malignancy. Whereas intense expression with distinct labelling of the cell borders was present in the epithelium of low-risk tumours, less intense expression of AQP 3 with alternating AQP-positive and AQP-negative tumour cells was found in intermediate-risk tumours. Extensive AQP-negative compartments and abrupt transition from strongly labelled to unlabelled tumour cells were evident in tumours of high malignancy. Complete loss of expression of AQP 3 was found in one third of high-risk cancers. AQP 4 was expressed in half of the tumours tested, and expression was independent of the ISUP/Gleason grade groups. Likewise, heterogenous expression of AQP 5 and AQP 7 was found in all tumours independent of malignancy grades. Finally, AQP 9 was not detected in malignant prostatic tissue. Representative results of these studies are shown in Fig. 4.

### Correlation of mRNA expression of AQP 3, AQP 4, AQP 7 and AQP 9 with clinicopathological parameters

**Fig. 5:**
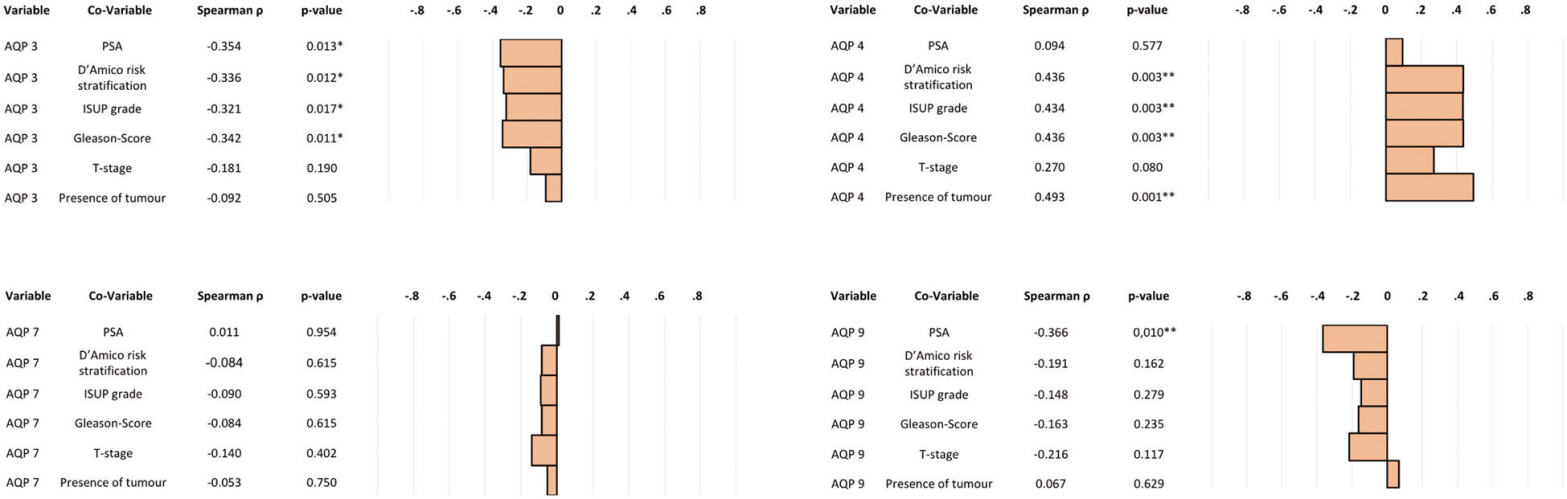
Spearman correlation of mRNA expression of AQP 3, AQP 4, AQP 7 and AQP 9 with clinicopathological parameters

AQP transcript expression of human prostate specimen was quantified by qPCR. The non-parametric Spearman’s rank correlation indicated a negative, statistically significant correlation between mRNA expression of AQP 3 and PSA levels (ρ = − 0.354; *p* = 0.013), D’Amico risk stratification (ρ = − 0.336; *p* = 0.012), ISUP grade (ρ = − 0.321; *p* = 0.017) and Gleason score (ρ = − 0.342; *p* = 0.011). Expression of AQP 4 revealed a statistically significant positive correlation with the presence of tumour (ρ = 0.493; *p* = 0.001), D’Amico risk stratification (*ρ* = 0.436; *p* = 0.003), ISUP grade (ρ = 0.434; *p* = 0.003) and Gleason score (ρ = 0.436; *p* = 0.003) but not with PSA (ρ = 0.094; *p* = 0.577) and tumour stage (*ρ* = 0.270; *p* = 0.080). By contrast, there was no correlation between AQP 7 mRNA expression and any clinicopathological parameter investigated. Expression of AQP 9 transcripts was found to be negatively correlated with PSA (ρ = − 0.366; *p* = 0.010). Please refer to Fig. 5.

## Discussion

The expression and function of AQPs has been investigated in the majority of human tissues [9]. Previous findings have suggested that fluid reabsorption and secretion in numerous organs are modulated by AQPs. However, human prostate tissue has not yet been systematically analysed in relation to AQP channel expression. The objective of the present study was to systematically investigate the expression and localisation of AQPs not only in human prostate (normal and malignant) cell lines in vitro, but also to investigate AQP expression in surgical specimens of BPH and PC of various malignancy grades.

Our study is the first to characterize human prostate tissue in relation to all 13 members of the AQP family. Transcripts for AQP 1, 3, 4, 7, 8, 10 and 11 were consistently detected in all four cell lines, while there was differential expression of AQP 5, 6 and 9. AQP 0, 2 and 12 were not detected in our study. Our mRNA transcript findings were corroborated at the protein level by immunofluorescence microscopy, where we detected AQP protein expression for those AQP family members where commercially available antibodies were able to be used. Our mRNA and protein findings in vitro are collectively in agreement with previous studies, which showed expression of AQP 1, 3, 5 in the human prostate at both the transcript and protein levels [7, 15]. Having characterized AQP expression in normal and malignant human prostate cell lines, we then investigated AQP expression in human BPH and PC specimens of various tumour grades via qPCR and immunohistochemistry.

The expression of AQPs by human prostate tissue and recent reports on their possible role in carcinogenesis in epithelial tissues raise interesting questions about the potential functional (biological) significance of AQPs in the development and progression of PC. We contend that it is particularly important to investigate whether the pattern (and localisation) of AQP expression could be of prognostic and/or of therapeutic value. Our results provide strong presumptive evidence that there is a correlation between the loss of AQP 3 expression and increased PC tumour grade. To our knowledge, this is the first report of progressive loss of AQP 3 expression in high-grade PC. Previous investigations into the significance of AQPs in non-urological tumours have almost invariably shown overexpression of AQP 3 and it has been hypothesized that AQP 3 may be a promising drug target in the treatment of various epithelial tumours [16–19]. The contrasting expression pattern of AQP 3 with intense labelling of AQP 3 in the case of BPH and well-differentiated PC, but non-homogeneous expression or loss of AQP 3 in the case of high-risk tumours, is noteworthy and may reflect tumour heterogeneity. Although this does not completely corroborate with cell line data, it is well known that in vitro cell lines do not necessarily reflect what happens in vivo. PC is well known for its tumour heterogeneity, which is reflected by diverse morphological manifestations and various molecular alterations associated with different tumour phenotypes [20].

With regard to clinicopathological parameters, downregulation of AQP3 was associated with higher preoperative PSA values, higher risk according to the D’Amico classification and a higher Gleason-/ISUP-grade. Interestingly, in support of our prostate cancer findings here, we previously reported a similar observation in urothelial carcinoma, with studies suggesting that the loss of AQP 3 may play a role in bladder cancer progression. We showed a significant correlation between AQP 3 protein expression and tumour stage and grade, with AQP 3 expression being reduced or lost in urothelial carcinomas of higher grade and stage [10, 21]. Furthermore, loss of AQP 3 expression was associated with worse progression-free and cancer-specific survival in patients with muscle-invasive bladder cancer [22].

The pro-tumorigenic effect of AQP 3 loss has also been reported for non-urological tumour entities. For instance, knockdown of AQP 3 expression resulted in increased migration and proliferation in gastric adenocarcinoma cell lines [16]. By contrast, overexpression of AQP 3 has been demonstrated for most other tumour entities, such as for squamous cell carcinomas [17]. Although establishing a functional role for AQP 3 expression in BPH and PC was beyond the scope of this study, our findings suggest that progressive loss of AQP 3 expression may be associated with worse clinical PC outcomes.

When studying AQP 4 expression, we found expression in half of carcinomas but not in any BPH specimens in immunohistochemistry. Moreover, a significant positive correlation was observed between mRNA expression in qPCR and D’Amico risk classification, Gleason- and ISUP-grading. Differential expression of AQP 4 in benign and cancerous tissue and its potential biological and clinical roles need to be elucidated in further studies.

AQP 5 and 7 were detected both in BPH and in PC specimens of all grades. However, there was no correlation of AQP 5 and 7 mRNA expression with any clinicopathological parameter in our cohort. These findings are similar to the observations made in a recent study by Park et al. [15]. Pust et al. previously reported highly variable AQP 5 expression in PC with both negative and intense expression of AQP 5 being linked to unfavourable outcomes [23]. In contrast to our findings, Li et al. demonstrated that AQP 5 expression was upregulated and associated with advanced stage, circulating tumour cells and inferior survival rates in PC [24]. These discrepancies and a wealth of somewhat contradictory results are interesting, and indicate that further studies are required to scrutinize the significance of AQP 5 expression in PC.

AQP 9 has previously been shown to be ubiquitously expressed along the male reproductive tract [25]. In concordance with this, we found expression of AQP 9 in normal prostatic cells in vitro and in BPH in vivo, whereas loss of expression was consistently demonstrated in cancer cell lines and in PC specimens. The demonstration of an inverse correlation of AQP 9 mRNA levels in qPCR and PSA supports this. Our study is the first to report the consistent loss of AQP 9 expression in PC, the biological significance of which still remains to be established. In hepatocellular cancer, for instance, decreased expression of AQP 9 resulted in increased resistance to apoptosis [26]. In rat prostates, AQP 9 expression has been shown to be regulated by androgens [8]. Similarly, Jiang et al. and Tian et al. suggested that expression of AQP 1, 3–8, 10–12 in PC is modified by androgens, at least in rats [27, 28]. However, there are little published data on its role in the human prostate. Hence, the potential role of AQP 9 in the carcinogenesis of PC needs to be addressed in future studies.

Despite the clear differential expression of AQP demonstrated in our study (particularly AQP 3), we do accept that our observational investigation was conducted on a relatively limited number of BPH and PC specimens. As such, despite their significance, our findings would certainly be strengthened by a bigger cohort of clinical specimens. One definite weakness of our study, however, is the lack of healthy prostate tissue serving as a control. Hence, we can only speculate on the relationship between AQP expression and function in normal human prostate tissue. Nevertheless, we did observe clear differential expression of AQP and a correlation with increasing malignancy in PC. While such an approach goes beyond the scope of the present study, future studies must certainly seek to prove a functional role for AQP 3 in PC progression. This would involve mechanistic studies and silencing with the RNAi-mediated knockdown of AQP 3 to assess biological endpoints relevant to tumour progression (proliferation, migration/invasion, and/or resistance to apoptotic stimuli) in the PC cell lines studied here. In addition, pre-clinical PDX models from patients with castration resistant PC might also be helpful. This would provide mechanistic insight into how changes in AQP expression may regulate tumour biology. We are currently addressing such questions, and are carrying out further studies that include sufficient patient numbers and long-term survival data to elucidate the potential clinical significance of AQP expression and its role in PC.

## Conclusions

This is the first study to demonstrate that several AQPs are expressed in human prostate cell lines, BPH and PC. Our results indicate differential expression of several AQPs in benign and malignant prostate tissue. A significant correlation was observed between AQP 3 protein expression and tumour grade, with a progressive loss of AQP 3 expression in more malignant tumours. However, it has yet to be determined whether AQPs play defined biological role(s) in the initiation and/or progression of PC and, more specifically, whether this could be of prognostic or therapeutic value.

## Additional files

Additional file 1: **Table S1.** RT-PCR oligonucleotide primers. Complete list of RT-PCR oligonucleotide primers used throughout this study. (DOCX 19 kb)

Additional file 2: **Table S2.** Antibodies used for immunofluorescence (IF) and immunohistochemistry (IHC) studies. Complete list of antibodies used for immunofluorescence (IF) and immunohistochemistry (IHC) throughout this study. (DOCX 17 kb)

## Abbreviations

AQP: Aquaporin
BPH: Benign prostatic hyperplasia
cDNA: Complementary desoxyribonucleic acid
DMEM: Dulbecco modified Eagle’s minimal essential medium
FBS: Fetal bovine serum
IF: Immunofluorescence
IHC: Immunhistochemistry
ISUP: International Society of Urological Pathology
mRNA: Messenger ribonucleic acid
PBGD: Porphobilinogen desaminase
PBS: Phosphate-buffered saline
PC: Prostate cancer
PDX: Patient derived xenograft
qPCR: Quantitative polymerase chain reaction
RNA: Ribonucleic acid
RPMI: Roswell Park Memorial Institute medium
RT-PCR: Reverse-transcriptase polymerase chain reaction
TNM: TNM Classification of Malignant Tumours
Tris-EDTA: Tris-ethylenediaminetetraacetic acid
UICC: Union internationale contre le cancer
