# Supplementary material for: Expression, localisation and potential significance of aquaporins in benign and malignant human prostate tissue
Source: BMC Urol. 2018 Sep 3;18:75. doi: 10.1186/s12894-018-0391-y (PMC6122723; doi:10.1186/s12894-018-0391-y)
Supplement: Supplementary file 1 — Table S1. RT-PCR oligonucleotide primers. Complete list of RT-PCR oligonucleotide primers used throughout this study. (DOCX 19 kb) [file 12894_2018_391_MOESM1_ESM.docx]

Additional file 1: Table S1 *RT-PCR oligonucleotide primers*

| Gene | Direction | Sequence (5´-3´) | Annealing-Temp. |
| --- | --- | --- | --- |
| AQP0 | Forward | TGTTCTGCAGGTGGCTATG | 65°C |
|  | Reverse | TGCTAGGTTTCCTCGGACAG |  |
| AQP1 | Forward | TCTGTAGCCCTTGGACACCT | 63°C |
|  | Reverse | CAAAGGACCGAGCAGGGTTA |  |
| AQP2 | Forward | GAGATCACGCCAGCAGACAT | 59°C |
|  | Reverse | GAAGAGCTCCACAGTCACCG |  |
| AQP3 | Forward | TGCTACCTACCCCTCTGGAC | 56°C |
|  | Reverse | GCCAGCACACACACGATAAG |  |
| AQP4 | Forward | TGGACCTGCAGTTATCATGGG | 56°C |
|  | Reverse | CAGCGAGGACAGCTCCTATG |  |
| AQP5 | Forward | CTGGCATCCTCTACGGTGTG | 59°C |
|  | Reverse | CCCTGCGTTGTGTTGTTGTT |  |
| AQP6 | Forward | TGCTCTGCTTTATGGGGTCA | 63°C |
|  | Reverse | GCCAGTTGAGACACTGTTCC |  |
| AQP7 | Forward | TTGGGTTTTGGCTTCGGAGT | 59°C |
|  | Reverse | CAGCGCACAGTTAGCAAAGG |  |
| AQP8 | Forward | CTTTGCCGTCACCGTGGATA | 59°C |
|  | Reverse | ACCACCGCAGGTCCAAAAG |  |
| AQP9 | Forward | TGAGTTCTTGGGCAGGTTCA | 56°C |
|  | Reverse | ATGACCCCTCCAAAACGTCC |  |
| AQP10 | Forward | GCACTGGGATGCTGATTGT | 65°C |
|  | Reverse | CCAGCCACGTAGGTGAAGAG |  |
| AQP11 | Forward | GACGCTGACGCTCGTCTACT | 65°C |
|  | Reverse | TCTGTGATGACCGCTTTGAG |  |
| AQP12 | Forward | GAACCTGTTCTACGGCCAGA | 63°C |
|  | Reverse | GTTCCAGGGTCCAGCTACAA |  |
| ß-Aktin | Forward | ATCATGTTTGAGACCTTCAA | 63°C |
|  | Reverse | CATCTCTTGCTCGAAGTCCA |  |
| PBGD | Forward | CCAGCTTGCTCGCATACAGA | 59°C |
|  | Reverse | ACACTGTCCGTCTGTATGCG |  |
