# Supplementary material for: Expression, localisation and potential significance of aquaporins in benign and malignant human prostate tissue
Source: BMC Urol. 2018 Sep 3;18:75. doi: 10.1186/s12894-018-0391-y (PMC6122723; doi:10.1186/s12894-018-0391-y)
Supplement: Supplementary file 2 — Table S2. Antibodies used for immunofluorescence (IF) and immunohistochemistry (IHC) studies. Complete list of antibodies used for immunofluorescence (IF) and immunohistochemistry (IHC) throughout this study. (DOCX 17 kb) [file 12894_2018_391_MOESM2_ESM.docx]

Additional file 2: Table S2: Antibodies used for immunofluorescence (IF) and immunohistochemistry (IHC) studies

| **Antibody** | **Host** | **Antigen** |  | | **Concentration (µg/ml) or ratio** | | **Source** |
| --- | --- | --- | --- | --- | --- | --- | --- |
|  |  |  |  | **IF** | | **IHC** |  |
| Anti-AQP 3 | rabbit | Human AQP 3 |  | 1:250 | | 1:1000 | Abcam, ab125219 |
| Anti-AQP 4 | rabbit | Human AQP 4 |  | 1:100 | | 1:100 | Santa Cruz, sc-20812 |
| Anti-AQP 5 | rabbit | Human AQP 5 |  | 1:50 | | 1:100 | Abcam, ab92320 |
| Anti-AQP 7 | rabbit | Human AQP 7 |  | 1:1000 | | 1:1000 | Abcam, ab85907 |
| Anti-AQP 9 | rabbit | Human AQP 9 |  | 1:100 | | 1:500 | Abcam, ab84828 |
